# Performable Case Studies in Ethics Education

**DOI:** 10.3390/healthcare5030057

**Published:** 2017-09-12

**Authors:** Richard Robeson, Nancy M. P. King

**Affiliations:** 1 Department of Communication, and Bioethics Faculty, Graduate School of Arts and Sciences, Wake Forest University, Winston-Salem, NC 27109, USA; robesor@wfu.edu; 2 Department of Social Sciences and Health Policy, Wake Forest School of Medicine, Center for Bioethics, Health and Society and Graduate Program in Bioethics, Wake Forest University, Winston-Salem, NC 27109, USA

**Keywords:** medical humanities, bioethics, case studies, interdisciplinary teaching, ethics teaching, readers’ theater, narrative medicine

## Abstract

Bioethics education often includes the study of short stories, novels, plays, and films, because such materials present case examples that can highlight relevant issues and questions especially vividly for a wide range of students. In addition, creative writing is widely used in the education of health professional students and in continuing education settings for health professionals. There are very few academic or professional disciplines that do not use case studies, but the case study in dialogic form has not been standard practice for thousands of years. Dramatic arts casuistry—the creation and performance of short case studies designed specifically to raise bioethics issues for discussion—represents an application of literature and the medical humanities that is both unique and uniquely valuable. This essay describes the development and history of a course that has been successfully taught to medical students and graduate bioethics students, in which the class researches, writes, and performs a case study designed to elicit reflection and discussion about a topic and set of bioethics issues of current interest to both academic and general audiences. The model is also suited to the presentation and discussion of existing case studies, both live and via on-demand audio.

## 1. Introduction and Background

In the Platonic Dialogue “Euthyphro” [1], the title character, on his way to court to prosecute his own father for impiety, by chance encounters Socrates, who presses Euthyphro to explain his unwavering certainty of the defendant’s guilt. Euthyphro’s response—namely, that there’s only one way to interpret the evidence—causes Socrates to ask a question that is, so to speak, the center of gravity of the Dialogue: even if there is only one way to read the evidence, a proposition which is extremely doubtful, by what mental, spiritual, or intellectual apparatus can you be certain that you know that one undeniably correct interpretation? This is a fundamental question that attends ethical issues, and it has remained so in the more than three thousand years since the Dialogues were composed; even as our science and our scientific rhetoric have become ever more sophisticated. The Platonic Dialogue, and especially the “Euthyphro,” is in many ways the ideal model for what we call the Performable Case Study (PCS); that is, a case study in dialogic form: between the poles of moral absolutism and moral relativism, there exists the opportunity to interrogate an ethical issue from more than one perspective, without succumbing to what journalist and author Eric Alterman calls “on-the-one-handism” [2]. This paper describes the development and use of performable case studies in bioethics education in North Carolina, both inside and outside academia.

In 1989–1990, the Department of Social Medicine, University of North Carolina (UNC)-Chapel Hill School of Medicine, introduced a radically new pedagogical strategy into its preclinical curriculum for second-year (MSII) medical students. “Medicine and Theater” engaged issues in biomedical ethics, research ethics, the physician–patient relationship, and related topics through the development and presentation of dramatic literature.

Medical and other health professional education has long incorporated ethics teaching in both the classroom and clinical settings. In the 1970s and 1980s, education in the medical humanities—including, for example, history and literature—began to augment medical ethics education as a means of increasing students’ sensitivity and responsiveness to the needs and interests of patients. The Literature and Medicine arena of the medical humanities makes sophisticated use of literary genres deemed noteworthy by virtue of the authors’ renown or topical currency, including fiction, creative nonfiction, pathography, patient histories, and even case law, statutes, scholarly bioethics literature, and informed consent documents.

Discussion of fiction and poetry, plays, and films addressing bioethics issues has been standard fare in health professional education for many years. For example, New York University (NYU)’s Literature, Arts, and Medicine database [3] began in 1993 and provides extensive information and valuable resources for use in teaching. Short stories, such as the physician-poet William Carlos Williams’ “The Use of Force” [4], and films like *Whose Life is it Anyway?* [5], *Wit* [6] (both of which were originally stage plays), *GATTACA* [7], *The Doctor* [8], and many others, have become standard educational fare in many universities and medical schools, as have novels such as *Never Let Me Go* [9] as well as new works like *Internal Medicine* [10]. Collections such as the *Social Medicine Reader* [11] include short stories, creative nonfiction essays, and poetry chosen to stimulate the discussion of bioethics issues and questions in the classroom. Collections of adapted [12] and original [13] short readers’ theater pieces have been published. Moreover, students today often write their own short stories or poetry for a literary magazine or bioethics blog, and many such literary outlets are based in medical, graduate, and even law schools. Indeed, writing narratives about “critical incidents”—difficult or otherwise memorable clinical experiences—has become quite common, not only during clinical education, but as an exercise for practicing physicians, nurses, and other health care providers [14].

“Medicine and Theater” was unique, however, in requiring classes of medical students to create an original dramatic work (case) to both reflect and provoke the careful argument required in bioethics analysis and pedagogy. In 2010, we brought the Medicine and Theater model to the Graduate Program in Bioethics at Wake Forest University (WFU), and, in a modified form, to undergraduate students in the Department of Communication. Now called “Performable Case Studies”, this course model engages students from a broad range of disciplines in researching, writing, presenting, and discussing short case studies in dialogic form that are designed to provoke thoughtful consideration and discussion.

When students create dialogic case studies, they learn to employ these genres to pose questions to audiences and each other through creation and performance. Engaging at once in analysis, synthesis, performance, and critical reflection opens up a range of perspectives on bioethics issues that no other type of course can achieve alone. Dramatic arts casuistry, as we have termed the process as a whole, thus moves well beyond any other mode of ethics inquiry as a means of engaging students in sustained and creative moral reflection.

To understand drama as a means of promoting public reflection and discourse on important societal questions is to return to a theatrical model that places the dramatic dialogue at or near the beginning of literary casuistry, which we know today through the plays of the Greeks, including Aeschylus, Aristophanes, Euripides, and Sophocles, as well as philosophic works, such as the Dialogues of Plato. What is forgotten in contemporary education is that these plays were intended to be accessible and to stimulate public engagement. It should not be surprising that a department called “Social Medicine”, devoted to a multidisciplinary approach to educating medical students to understand the social and cultural context of health care by means of the medical humanities and social sciences, would recognize the value of a course such as Medicine and Theater. Nonetheless, the disciplinary segregation and sequestration of expert knowledge that has characterized academia in recent centuries (i.e., increasing specialization, or “siloing”) has rendered many universities (and students) skeptical about the possibility and value of such a course. Only recently has the resurgence of interest in creativity studies, interdisciplinary teaching and scholarship, and the importance of narrative begun to stand against this long history [15]. Even multidisciplinary fields, such as bioethics and communication, may at times need reminders that the arts and humanities play an essential role in teaching and learning about health, illness, and health care [16].

## 2. History of Performable Case Studies

What is now Performable Case Studies began life in 1988 as an extracurricular enrichment activity for medical students at UNC-Chapel Hill, funded by the North Carolina Humanities Council (NCHC) as a community outreach activity, designed to stimulate engagement and discussion between health care professionals and students and the general public about a broad range of ethical and social issues arising from the physician–patient relationship and the role of medicine in society. The first “staged reading” was an adaptation of William Carlos Williams’ short story “Mind and Body” from the collection *The Doctor Stories* [17]. This collection of short fiction about medicine, by an acclaimed poet who was also a physician, is frequently used in medical student teaching. The stories are spare in style and highly evocative. In addition, they are primarily dialogue, and therefore easy to adapt.

The presentation model used in the first project has persisted: a short performance followed by a discussion between the readers and the audience, moderated by a scholar in medical humanities or bioethics. The staged reading format, in which the performers read rather than memorize their lines and minimal use is made of stage movement and props, was explained in a “curtain speech”, as was the integral role of the post-performance discussion. “Mind and Body” was performed first at the UNC Student Union and subsequently in a popular Chapel Hill coffee shop with a performance area. It was also adapted for radio, produced and directed by Robeson, with a cast of professionals, and broadcast in 1990 on ”Soundings”, a nationally syndicated radio program emanating from the National Humanities Center in Research Triangle Park, North Carolina (NC), followed by a discussion with physician–ethicist Dr. Eric Cassell about the physician–patient relationship [18].

“Mind and Body”—a challenging and ambiguous story in which a physician tries, with uncertain success, to understand and meet the needs of a new patient—spurred vigorous audience discussion. It was abundantly clear that this presentation model worked for audiences. Williams’ publisher, after seeing the text of the adaptation and the videotape of the Student Union performance and discussion, was, moreover, delighted to provide a broad permission to adapt the stories in *The Doctor Stories* for educational uses.

“Mind and Body” also made clear how well the performable case studies model worked for the health professionals and students serving as readers, in three ways. First, because the staging was minimal and no memorization was required, rehearsals could easily incorporate discussion. The readers thus undertook their own exploration of the characters and the story, examined different points of view about the issues addressed, and developed questions they wished to discuss with audiences. Second, the readers, like actors everywhere, had the opportunity to examine characters from the inside, and often gained significant insights from portraying someone like the patient in the story. And third, discussing the same reading with different audiences—especially after having discussed it among themselves in rehearsal—always resulted in valuable new mutual learning.

The success of the first performable case study led to a collaboration between NCHC and UNC-Chapel Hill (both the medical school and the graduate school), East Carolina University (the medical school), and Duke University (the medical school), in a successful application to the National Endowment for the Humanities for a 3-year Exemplary Award (1990–1992) to provide support for traveling readers’ theater companies from the participating universities. A repertoire of case studies, adapted from the 20th century literary canon, was developed by artistic directors from each university, and a coordinator helped to advertise, plan, schedule, and facilitate performance/discussion sessions on evenings and weekends around the state. The venues included public libraries; area colleges, community colleges, and universities; medical society meetings; and retirement communities. NCHC, upon receiving notice of the success of the application, also learned that this project was considered the most innovative of any during that funding cycle, hence the designation “Exemplary”.

After several years of pursuing performable case studies as a means of addressing medical ethics issues solely in an extracurricular capacity, particularly with students, faculty, and in some cases staff of the medical school as readers, the time was ripe to incorporate the model into the curriculum for medical students. And, once the outreach model became a part of the curriculum as “Medicine and Theater”, it was soon apparent that the model’s pedagogical value could be enhanced by working from original sources rather than creating adaptations from the canon. Adaptations must begin from predetermined narratives, which also typically offer a limited and predetermined range of interpretations. The work of identifying differing lines of ethical inquiry when creating an original case study, particularly when even the facts of the case may be in dispute, is by definition more intellectually and creatively demanding.

Since 1988, Richard Robeson, working alone, with medical or graduate students, or in collaboration with Nancy King and other colleagues, has created more than two dozen performable case studies, almost all of which have been presented as staged readings, with discussion, in a variety of classroom and community settings. This body of work includes materials as diverse as adaptations of existing scripts (e.g., George Bernard Shaw’s *The Doctors’ Dilemma* [19]; Henrik Ibsen’s *An Enemy of the People* [20]), adaptations of short stories (e.g., numerous works from William Carlos Williams’ collection *The Doctor Stories*, as well as stories by Wendell Berry and Perri Klass); and original works based on court cases and news stories raising significant bioethics issues, such as the activities of Dr. Jack Kevorkian; organ transplantation; medical and research decision-making; and environmental justice. These works have been developed and used in the classroom, as medical grand rounds, in public humanities projects, as educational materials in public health research, as radio readings, and in conference settings.

The “Medicine and Theater” course confronted a wide range of topics, from taking a fresh look at some of bioethics’ earliest issues (e.g., the Tuskegee syphilis study) to some that remain emblematic of the discipline’s concerns (e.g., surrogate decision-making; faith-based treatment refusal) to others that it is only beginning to consider (e.g., ethical issues in sports medicine). Its subsequent iteration since 2010 as “Performable Case Studies” in the Graduate Program in Bioethics at Wake Forest University has addressed a similarly varied set of issues through case studies involving, for example, military research, gene patenting and genetic testing, and the nonconsensual drug testing of pregnant women.

## 3. Process and Products

The PCS model is in some respects a laboratory, in others a workshop. Although the topics and subject/issue areas change with each semester’s course offering, the process by which cases are developed has remained consistent since the early 1990s. Case development is accomplished during a single 12–14-week semester in three phases of roughly equal length, described in the course syllabus as follows:

Phase One: Discussion and Analysis. This is approximately five class sessions. We will discuss and analyze the contextual and background material, and the perspectives represented by stakeholders, scholars, and reporters. During this phase, we will identify lines of bioethical inquiry, which in turn will inform (1) students’ targeted additional research; and (2) the creation of principals (characters) in the case to be constructed.

Phase Two: Targeted Research. This is approximately three class sessions. Each student will do research specifically intended to inform and advance the work of constructing the case, present their research and analysis to the rest of the class, and field questions regarding the research itself and its applicability to the bioethics framework. Additional presentation time may be necessary, depending upon the final class size.

Phase Three: Case Study Construction (Writing). It is intended that this Phase take place entirely during class sessions, and will cover the remainder of the semester. At this juncture, characters have been revealed via the framing of bioethics issues. The engagement of characters with each other and their circumstances (e.g., patients-subjects, healthcare providers/researchers, family members, investors, reporters, etc.) will be the substance of the case study. The class will divide into writing teams, according to which characters interact in a given setting, in a given class period. These interactions will be facilitated by the faculty instructor. Each session of Phase Three will begin with a review of the previous session’s writing, and will end with a review of the current session’s writing [21].

The writing process, which begins approximately two-thirds of the way into the semester, is facilitated via the intra- and inter-group dynamics of as many as four writing teams, whose memberships change according to the scene, dialog, and character interactions of a given writing session. Circulating between writing teams during class sessions, the faculty instructor is able to work with each team, providing on-the-spot monitoring, prompting, and editorial suggestions, both to ensure narrative continuity and to maintain a proper balance between character, narrative, and issues. Scripts are crafted to highlight questions and tensions, in order to promote lively and nuanced post-performance discussion. The class prepares lists of audience discussion questions and drafts framing materials (event introduction, and guidelines for discussion facilitators) to ensure that all participants understand the necessary complementarity of performance and discussion. Every aspect of every reading thus points toward discussion. Moreover, discussion is designed in the course and managed in each presentation not only to build on the case presented, but also to incorporate the experiences and perceptions of diverse audiences, whose insights have the capacity to greatly enrich the interpretation and discussion of the issues.

One of the premier advantages of this approach to bioethics/medical humanities pedagogy is its usefulness in engaging students from a broad range of educational and disciplinary backgrounds. An MSII class, for example, will necessarily represent strong backgrounds in the life sciences, even if some might have had a humanities or social science discipline as their major undergraduate course of study. The Graduate Program in Bioethics at Wake Forest University, however, not only shows a similar breadth of disciplinary backgrounds, but also has a student population that includes fully credentialled physicians, nurses, physician assistants, and practicing attorneys, as well as current and future medical, law, and divinity students, and others with undergraduate backgrounds in STEM disciplines as well as the humanities and social sciences. Although there are often significant differences in disciplinary background and career experience within a given class, or from one class to another, the learning objectives for students are consistent, regardless of topic or issue area. The PCS syllabus states these objectives as: (1) Careful and thorough research. (2) In-depth analysis and discussion, with a particular eye/ear toward implied meanings or interpretations; and a continual alertness to the possibility of the existence of a prior question, and a willingness to raise it—or to have it raised to you—if such be the case. (3) Clearly expressed and well-rendered arguments in written and oral form. (4) The ability to craft a complex but succinct narrative with no deliberate intent to bias how the narrative will be interpreted. (5) The ability to work collaboratively.

It is expected neither that everyone should agree on all points at a semester’s beginning, nor that everyone should agree at the semester’s end. Furthermore, it is not required that the case itself represent the homogenization of the perspectives and analyses that emerge as the semester unfolds. Rather, it is understood that divergences of opinion and interpretation will enrich not only the process but also the end result.

This pedagogical method works equally well with all student cohorts, not only because of the scholarly imperatives that attend bioethics inquiry regardless of method, but also because this particular method, as mentioned previously, allows (even demands) a recognition that complex ethical issues are not binary, but multifaceted.

Professor Robeson and his students have researched and written 13 original case studies to date. Each of those created by bioethics graduate students has been presented, with discussion, as a campus-wide event sponsored by the Wake Forest University Center for Bioethics, Health, and Society. In addition, in 2011, “The Burial Society”, a PCS about the Tuskegee Syphilis Study that was created in the Medicine and Theater course in 1994, was presented [22] as part of a scholarly visit by James Jones, to commemorate the 30th anniversary of the publication of his classic historical analysis *Bad Blood* [23]. Students in the Department of Communication have also read, studied, and analyzed several performable case studies written in previous years, and then made audio recordings under Professor Robeson’s direction.

One example of the breadth and versatility of this creative process is provided by “Whose Right?—A Neurofamilial Tangle”. The case that formed the basis of the work of the class was a family controversy that arose when the wife of a retired physician with dementia allegedly consulted Dr. Jack Kevorkian on her husband’s behalf. The couple’s adult children divided over what was best for their father, and a number of legal proceedings ensued.

The class was provided by Richard Robeson with newspaper clippings detailing the court proceedings and interviews with family members. Nancy King compiled resources about end-of-life decisions, capacity to consent, surrogate decision-making, and the distinctions between withholding treatment, physician-assisted suicide, and euthanasia. Under Professor Robeson’s guidance, the students wrote a performable case study that, by exploring family dynamics, raised important questions about how to interpret patients’ wishes; the relationship between autonomy and best interests, and different interpretations of each; the complex entanglement between emotion and reason in family decision-making; and the limits of the law in end-of-life issues.

The case study, while short, is vivid and surprisingly nuanced. This is attributable in no small measure to the aforementioned tri-phasic process. By the time that the case construction is begun, the students, having spent two-thirds of a semester identifying and discussing the salient ethical issues, have also identified (or imagined) stakeholders who represent those issues. From this point, the essential writing task is to engage the issue or circumstance *as the stakeholder*. It is often the case that a student’s individual understanding of the issues is quite at odds with those of the stakeholder the student has elected to represent. Given that most of the students who have taken this course over the years expect to work in clinical ethics or biomedical caregiving settings, the ability to identify with a challenging or unfamiliar ethical position has both immediate and enduring value. The writing teams vary from one class session to another, depending upon which elements of the narrative arc are being developed.

It is also critically important that the lines of dialogue be spoken aloud as they are written. Having the dialogue develop as spoken language helps to ensure that it is conversational, and embodying the character can sometimes result in exchanges that are completely unplanned.

“Whose Right?—A Neurofamilial Tangle”, though based upon a well-documented court case during the height of Dr. Jack Kervorkian’s infamy [24], focuses less upon the litigation than upon the dynamics within a family—in which the patient and three of his four adult children are physicians, and the fourth is married to a physician—who have drastically differing views on the scope and meaning of spousal loyalty, and the precise nature of the duty to one’s parents. As Evelyn, the mother, conflicted over a decades-old promise made to husband Phil, confides in Karen, her non-physician daughter [25]:

EVELYN: Karen, I’m just not sure what to do anymore. Taking care of your father has become so difficult. I’m really torn between what I know he wants and wishing things could be the way they were before. But I know they can’t. He won’t get better ... only worse ....

KAREN: Maybe we should think about getting a nurse to help you look after him.

EVELYN: It’s not that I mind taking care of him ... that wasn’t actually what I was thinking of.... I’ve been wondering about this doctor in Michigan ... this doctor who ....... helps people who are terminally ill. Dr. Rubenstein. Have you heard of him?

KAREN: Mom, you can’t be serious. In the first place, Dad’s not terminally ill

...

EVELYN: A long time ago, I made a promise to your father. I promised him I wouldn’t let his life end like your grandmother’s did. I think the time has come to make a decision.

KAREN: You’ve already contacted Dr. Rubenstein, haven’t you?

EVELYN: Not yet.

The dialogue here emerged from the positions that the students determined were held by these two stakeholders, based on their research, discussion, and writing work. In the classroom, where other writing teams are working at the same time, similar exchanges are taking place. Thus, the rest of the class is unaware that these lines are being developed, until the end of the period, when all of the session’s work is shared by being read aloud.

The perceived ethical imperatives among family members are sufficiently distinct that the moral high ground, as it were, is difficult to discern. And this, of course, is the reason that a case study in this form has such power, both as narrative, and as stimulus to multiple lines of bioethical inquiry. For example, Hank, in denouncing his mother for even entertaining the idea of physician-assisted death for his father, irrespective of any pact they made decades ago, implies an existing animus toward his mother, which also raises a question: to what if any extent are his reasons relevant to what he seeks to accomplish, namely, preserving his father’s life [26]?

EVELYN: HANK: Mom, I’m really concerned about Dad. I just got done talking to him and he seems suicidal!

EVELYN: What do you mean?

HANK: You wouldn’t believe the things he says: “I don’t want to go on like this”; “My life isn’t worth living.”

EVELYN: Hank, these things aren’t new. Why are you surprised?

HANK: I’m not surprised at all at him! I’m surprised at you. How can you just sit there like I just said “The soup’s ready”? I just told you your husband wants to kill himself and I think you should be doing more.

EVELYN: I’m doing the best I can. You aren’t here. You don’t see him. He said before he got sick that he’d rather die than live the life he’s living now.

HANK: And you support his taking his own life?

EVELYN: Well, in his condition, it’s unlikely he could.

HANK: You wouldn’t actually consider helping him, would you?

EVELYN: [Looks down.]

HANK: Well, would you?

EVELYN: You don’t understand -- Every day I take care of him. It’s so hard ....

HANK: Hard? Hard for you? What about him? What about us? If it’s so hard, why don’t you just call up Dr. Rubenstein and have him put Dad out of *your* misery?

EVELYN: [Fidgets.]

HANK: I can’t believe you’re are actually considering it.

EVELYN: I know what your father would have wanted.

HANK: To kill himself?!! Or should I say, to have you kill him? My God, Mom, how can you be so heartless? My father, your husband, is sick; and one-day-too-many going to the grocery store for him and you decide to kill him! Life is God’s greatest gift, Mom; and no ideal, however glorious or however selfish, is worth the taking of it AND IF YOU THINK there’s a chance in hell you’d get away with it, you’ve got ANOTHER THINK COMING!

In this, as in all Performable Case Studies, the ending does not resolve the conflicts or ethical conundrums. A case study in whatever form can be considered to most properly be the beginning of a discussion, rather than a declaration of a certain point of view. The dialogic form makes multidimensionality much more readily available, and subsequently carries with it its own ethical imperative to fully exploit this capability.

In addition to its performance by the Medicine and Theater students in 1997, “Whose Right?” was also performed by bioethics scholars and teachers from across the nation at the 1999 Bioethics Summer Retreat, in 2002 by members of the Charlotte, NC-based Bioethics Resource Group, and in 2008 by Wake Forest University law students. Discussions in each instance focused not only on the issues themselves, but also on the pedagogical uses of the performable case study and the relationships between the issues depicted in the case study and the experiences and perceptions of the readers and audiences.

Responses and reactions from audiences during discussion have been varied, and continue to be so, according to the backgrounds and disciplinary predispositions of both academic and outreach audiences. The thesis questions that inform the development of each PCS and the questions that prompt discussion following presentations are not intended to elicit particular responses. For example, “Unquantifiable Risk”, a PCS that inquires into xenotransplantation as a possible source of organs for human transplantation, also raises the question: what do we mean when we say that there is an “organ shortage”? Likewise, a readily available point of departure in discussing the Tuskegee Syphilis Study is the undeniable presence of an institutionally racist agenda, not to mention a highly suspect (at best) research question. But quite a different vein of analysis is available if the thesis question, and thus the development of the case study and planning for audience discussion, concerns what the Tuskegee subjects have in common with clinical research subjects in general.

In addition, many students in the course have chosen to examine additional aspects of the case study they helped to build through creative writing in their final papers, thus continuing to explore case issues such as the complexity and ambiguity of family decision-making about the end-of-life issues addressed in “Whose Right?”.

The feasibility and effectiveness of the performable case study model can be extended to additional settings, both in and outside the classroom, by essentially working backwards: first engaging participants in a concert reading, then discussing the issues addressed by the case study, how the theatrical format influences identification and discussion of the issues, and the relationship between research into scientific, policy, and ethical issues and their creative use in writing case studies. (See Table 1 for a complete list of performable case studies, including their creation and uses.) For example, the case study “Unquantifiable Risk” addresses issues raised by the prospect of xenotransplantation, including: the balancing of risks of harm and potential benefits; the uncertainties of first-in-human xenotransplantation; the blurring of lines between research and treatment; and the processes of recruitment and consent when patients are also research subjects. The case study was researched, developed, and presented by Professor Robeson and MSII students at UNC-Chapel Hill in 1997–1998, and studied and recorded by students in the Department of Communication at WFU in 2012. The issues posed have not changed in the intervening years.

## 4. Conclusions

The PCS model grew out of recognition of the value of case-based teaching in the multidisciplinary field of bioethics, combined with a commitment to the intersection of art and scholarship, and shaped by the imperative of fostering effective communication between health care professionals and the public. Bioethics pedagogy, increasingly popular both in and beyond health-related education, is itself eclectic. At its best, it makes use of a variety of disciplinary perspectives, though there is a general tendency to prioritize philosophy and Western classical philosophical reasoning. What is most important about bioethics education—about any ethics education, for that matter—is its inculcation of habits of critical moral reflection, especially the ability and willingness to question one’s own viewpoint and to foster productive reflection and discussion in others. Yet much education involving literature and narrative falls short of promoting critical reflection. Instead, the author’s perspective is thoughtfully challenged only in rare circumstances; as a result, the narrator—whether doctor or patient—risks becoming unassailable, and the narrative risks becoming propaganda: that is, a work meant to disallow nuance or the possibility of alternate interpretations.

In signal contrast, the process of creating performable case studies from contemporary cases treats the work as a stimulus for critical reflection. Student writers develop characters whose voices are complex and whose viewpoints are plausible and will stand up to many-faceted discussion. Student readers gain insights from representing views different from their own, and grow to understand and appreciate others’ experiences, in at least a small way, through this process. Students also learn from discussion with audiences, both academic and non-academic. How the audience experiences the story—what they bring to it and what they take from it—is an essential component of the performance/discussion combination. Indeed, the PCS model is based on understanding the engagement with audiences as ethics education outreach for them, and as one component of ethics education for students in the course.

Catharsis, as Aristotle teaches us in the “Poetics”, is one of the principal currencies of dramatic art: an emotional release, “purgation” (sometimes translated as “purification” [27]) through “pity and fear” [28]. To the extent that distinctions between art and religion existed in the Greece of Aristotle, he acknowledges catharsis as an artistic conceit. A case study, by contrast, is a stimulus to critical reflection and analysis. And while a case study in dialogic form does not discourage or subvert an emotional connection to the facts or persons under consideration, its primary function is not—nor can it be—to induce a cathartic experience. This is a crucial distinction between a dialogic case study and expressly “theatrical” models, including reader’s theater.

Distinguishing the PCS model from traditional forms of theater is important for other reasons as well. First, it helps to democratize the PCS process and products, by making it clear that students and faculty both without and with theatrical experience and expertise can readily work together in producing, presenting, and discussing case studies. Second, it helps to reassure students who may at first not consider themselves sufficiently creative or extroverted to participate in the course. Hesitant students invariably come to appreciate the process and to take pride in the product.

The depth, scope, and flexibility of active engagement with ethics represented by this course provides a pedagogical model that can be adapted effectively for any ethics education curriculum; and that, in addition, incorporates community outreach that is both engaging and educational. Bioethics, or the ethics of health care and science, is a naturally attractive subject for dramatic arts casuistry because its case examples are vivid, complex, and compelling. However, many other manifestations of ethics in society are just as apropos, making this educational model extremely versatile. Any discipline, whether academic, professional, or outreach-based, that customarily utilizes the case study as a pedagogical, developmental, or organizational tool, can benefit from this model. Professor Robeson has employed it in public health and environmental justice contexts, and as part of a leadership training conference. Similarly important ethical issues arise—and are the focus of educational innovation—in business and entrepreneurship, in law and economics, and in laboratory science.

Just as ethics provides a means of approaching and discussing controversial issues without personal attacks, performable case studies provide a launching point for ethics discussion that can engage, explore, and even challenge multiple viewpoints while minimizing antipathy. It is important to keep in mind that the desired outcome is critical engagement with the issues that are raised, implied, or interrogated, regardless of whether the PCS model or another similarly open and creative teaching and learning method is employed.

Those studying moral philosophy, religion, psychology, political science, and many other areas also have reason to examine whether and how “everyday ethics” fits with the practical and political perspectives of those disciplines. And, in dramatic arts casuistry, all have the means to make an examination that is active, creative, nuanced, and productive.

## Figures and Tables

**Table 1:** Performable Case Studies 1988–2016. Subject/Issue Areas and Settings.

**Medicine And Theater—MSII, UNC Chapel Hill School of Medicine**		
Adaptations *		
**Year**	**Title (author)**	**Theme(s)**
1989–90	The Doctor’s Dilemma (G. B. Shaw)	Resource allocation
1990–91	Enemy of the People (Henrik Ibsen)	Public health/biohazard
1991–92	The Doctor and the Devils (Dylan Thomas)	Research ethics
Original **		
1992–93	The Dance of Power	*The Georgetown College Case* – faith-based treatment refusal
1993–94	The Burial Society	US Public Health Service Syphilis Study at Tuskegee University
1994–95	Alea Jacta Est (The Die is Cast)+	Dr. Christian Barnaard/the first successful human heart transplant
1995–96	Duty to Rescue?	*McFall vs. Shimp* – bone marrow donor refusal
1996–97	Whose Right? A Neurofamilial Tangle	Alzheimer’s disease/physician-assisted death
1997–98	Unquantifiable Risk	Xenotransplantation – imagining the first permanent animal-to-human organ transplant
1998–99	Thalidomide on Trial	The re-introduction of the teratogen for new medical uses
1999–00	Conscious Decision	Faith-based treatment refusal complicated by criminal charges of proximate cause
2000–01	Drawn and Quartered	Confluence of bioethics, high-profile intercollegiate athletics and gender equity
**NC Humanities Council/National Endowment for the Humanities Exemplary Award Project**		
Adaptations ***		
1988	Mind and Body (William Carlos Williams)	Trust, mistrust and empathy in the physician-patient relationship
1991	A Face of Stone (William Carlos Williams)	Overcoming physician prejudice
1992	Old Doc Rivers (William Carlos Williams)	Rural medicine, physician substance abuse
1993	That Distant Land (Wendell Berry)	Death and dying in a close-knit rural community
1994	Invasions (Perri Klass and Sarah Klemmer)	Patients, physicians and privacy in the hospital
**National Institute of Environmental Health Sciences/UNC School of Public Health—Exchange Project**		
Original ****		
2002	Lost: Sanctuary	Conservation, Environmental Justice, Eminent Domain
The same issue (disaster relief/public health) from three different ideological, disciplinary and stakeholder perspectives		
2003	A Certain Measure	Displaced flood victim
2004–05	Fall Toward Grace	Low-seniority county health dept. staffer
2005	A Matter of Engagement	Public health school academicians
**Performable Case Studies—M. A. in Bioethics, Wake Forest University**		
2011	Camera Obscura	Myriad Genetics, Inc., gene patenting, women’s health
2012	A Defining Error	*Ferguson v. City of Charleston*, involuntary drug screening/arrest at prenatal clinics
2015	Incident-To Service	*United States v. Stanley*, US Govt. hallucinogen experiments
2016	Perhaps A Level Field	Third-part litigation financing industry; unethical surgical innovation
**Communication Ethics/Bioethics: An Interface—Department of Communications/M. A. in Bioethics, Wake Forest University**		
2012	Unquantifiable Risk (For Electronic and Digital Media)	Xenotransplantation (cf. above), biotechnology, transhumanism
2013	Camera Obscura (For Electronic and Digital Media)	Myriad Genetics, Inc., gene patenting, women’s health

* Adaptations by Richard Robeson with Karyn Traut, students, and Nancy King. ** Performable case studies by Richard Robeson with students. *** Adaptations by Richard Robeson. **** Performable case studies by Richard Robeson. + Grand Rounds, Dept. of Family Medicine, UNC Hospitals.
